# Papillomavirus-Associated Tumor Formation Critically Depends on c-Fos Expression Induced by Viral Protein E2 and Bromodomain Protein Brd4

**DOI:** 10.1371/journal.ppat.1005366

**Published:** 2016-01-04

**Authors:** Maria Delcuratolo, Jasmin Fertey, Markus Schneider, Johanna Schuetz, Natalie Leiprecht, Benjamin Hudjetz, Stephan Brodbeck, Silke Corall, Marcel Dreer, Roxana Michaela Schwab, Martin Grimm, Shwu-Yuan Wu, Frank Stubenrauch, Cheng-Ming Chiang, Thomas Iftner

**Affiliations:** 1 Division of Experimental Virology, Institute of Medical Virology, University Hospital Tübingen, Tübingen, Germany; 2 Department of Oral and Maxillofacial Surgery, University Hospital Tübingen, Tübingen, Germany; 3 University of Texas Southwestern Medical Center, Simmons Comprehensive Cancer Center, Department of Biochemistry, Department of Pharmacology, Dallas, Texas, United States of America; National Institute of Allergy and Infectious Diseases, National Institutes of Health, UNITED STATES

## Abstract

We investigated the mechanism of how the papillomavirus E2 transcription factor can activate promoters through activator protein (AP)1 binding sites. Using an unbiased approach with an inducible cell line expressing the viral transcription factor E2 and transcriptome analysis, we found that E2 induces the expression of the two AP1 components c-Fos and FosB in a Brd4-dependent manner. *In vitro* RNA interference confirmed that c-Fos is one of the AP1 members driving the expression of viral oncogenes E6/E7. Mutation analysis and *in vivo* RNA interference identified an essential role for c-Fos/AP1 and also for the bromodomain protein Brd4 for papillomavirus-induced tumorigenesis. Lastly, chromatin immunoprecipitation analysis demonstrated that E2 binds together with Brd4 to a canonical E2 binding site (E2BS) in the promoter of c-Fos, thus activating c-Fos expression. Thus, we identified a novel way how E2 activates the viral oncogene promoter and show that E2 may act as a viral oncogene by direct activation of c-Fos involved in skin tumorigenesis.

## Author Summary

Human Papillomaviruses (HPV) are the etiological agents of cervical cancer and of skin cancer in individuals with the inherited disease epidermodysplasia verruciformis (EV). While the role of the viral oncogenes E6/E7 as drivers of tumorigenesis in cervical cancer has been firmly established, the contribution of the early viral genes in skin cancer is less clear. For EV-associated HPV8 and for the skin cancer model system using cottontail rabbit PV, an important role of the viral E2 protein in tumorigenesis was suggested earlier and regulation of cellular genes by E2 through different mechanisms was demonstrated. We show now that the viral E2 and cellular Brd4 act together to induce the cellular gene c-Fos, which as a member of the AP-1 complex, is involved in the regulation of cellular genes and the viral promoter driving the expression of viral oncogenes. As c-Fos has also been shown to be essential for skin cancer, E2 contributes to tumorigenesis via expression of E6/E7 as well as by increasing c-Fos.

## Introduction

Papillomaviruses (PV) are small double-stranded (ds) DNA viruses that are able to cause epithelial tumors, including cancers of the *cervix uteri* and the oropharynx and are likely to be involved in the development of non-melanoma skin cancer [1]. Expression of viral oncogenes E6 and E7 that dysregulate the cell cycle via direct interaction with the tumor suppressor proteins p53 and pRb, respectively, is controlled by the viral protein E2. Earlier results from us have shown that mutations in conserved amino acids of the trans-activation domain of E2, which is a regulator of viral transcription and replication, dramatically reduced tumor induction in our rabbit animal model system [2]. Those amino acids were later proven to be important for the interaction with Brd4, which belongs to the family of bromodomain- and extra-terminal (BET) proteins that are key regulators of transcription by controlling networks of genes, including P-TEFb and Mediator, involved in cellular proliferation and cell cycle regulation [3]. Dysregulation of BET protein activity has been linked to different cancers, notably NUT-midline carcinoma [4]. Papillomaviruses require Brd4 for efficient genome maintenance, partitioning and tethering viral genomes to the host chromosome in mitosis [5,6] and binding to Brd4 stabilizes the E2 protein [7–9]. Both the transcriptional activation and the repression function of E2 have been linked to an interaction of E2 with the far C-terminus of Brd4 [8]. Transcriptional repression of viral promoters controlling E6/E7 oncogene expression via E2 is partly due to the circumstance that P-TEFb and E2 compete for a binding site at the C-terminal domain (CTD) of Brd4, while P-TEFb in complex with Brd4 is required for promoter activation [10]. In addition, sterical hindrance of basal transcription factors, like TBP, through the binding of E2 to two binding sites in close proximity to the transcription start site plays a role in E2-mediated repression [5]. The mechanism of transcriptional activation involving the E2-Brd4 complex is, however, less clear. Trans-activation of the natural enhancer/promoter has so far only been described for bovine papillomavirus, cottontail rabbit papillomavirus [11] and cancer-associated EV papillomaviruses [5,12], while genital high-risk types involved in cervical cancer always have two E2 binding sites (E2BS) in close proximity to the transcription start site, which mediate repression of the promoter via E2. We have recently shown that PV E2 protein induces the mRNA encoding matrix-metalloproteinase 9 (MMP9) via the proximal AP1 site within the MMP9 promoter [13]. Interestingly, AP1 sites in the upstream regulatory region (URR) of PVs have been shown to be essential for the activity of the promoter driving expression of the viral oncogenes E6/E7 and to be responsive to stimulation by phorbol esters. Mutations of an AP1 site conserved in position in the enhancer of papillomaviruses almost completely abolished the activity despite the presence of intact E2 binding sites [14,15]. AP1 sites mediate transcriptional activation, when bound by dimeric complexes consisting of c-jun and c-Fos family members and the sequence composition of the AP1 sites determines the binding affinity of the 18 different dimeric AP1 complexes [16]. Interestingly, a shift in the AP1 complex composition from c-Jun/Fra-1 to c-Jun/c-Fos heterodimers has been observed during tumor progression [17,18] and c-Fos has been shown to be essential for malignant progression of skin tumors [19,20]. As the MMP9 promoter induction by E2 was independent of E2 binding sites, but required AP1 binding sites and interaction of E2 with Brd4 [13] we now investigated the mechanism how E2 can activate promoters via AP1 binding sites. In the present study, we identified a novel pathway how the E2/Brd4 complex activates the papillomavirus promoter via c-Fos and show that each of these two factors is essential for tumorigenesis.

## Results

### CRPV E2 transactivates TPA- but not cAMP-dependent reporters

**Fig 1:**
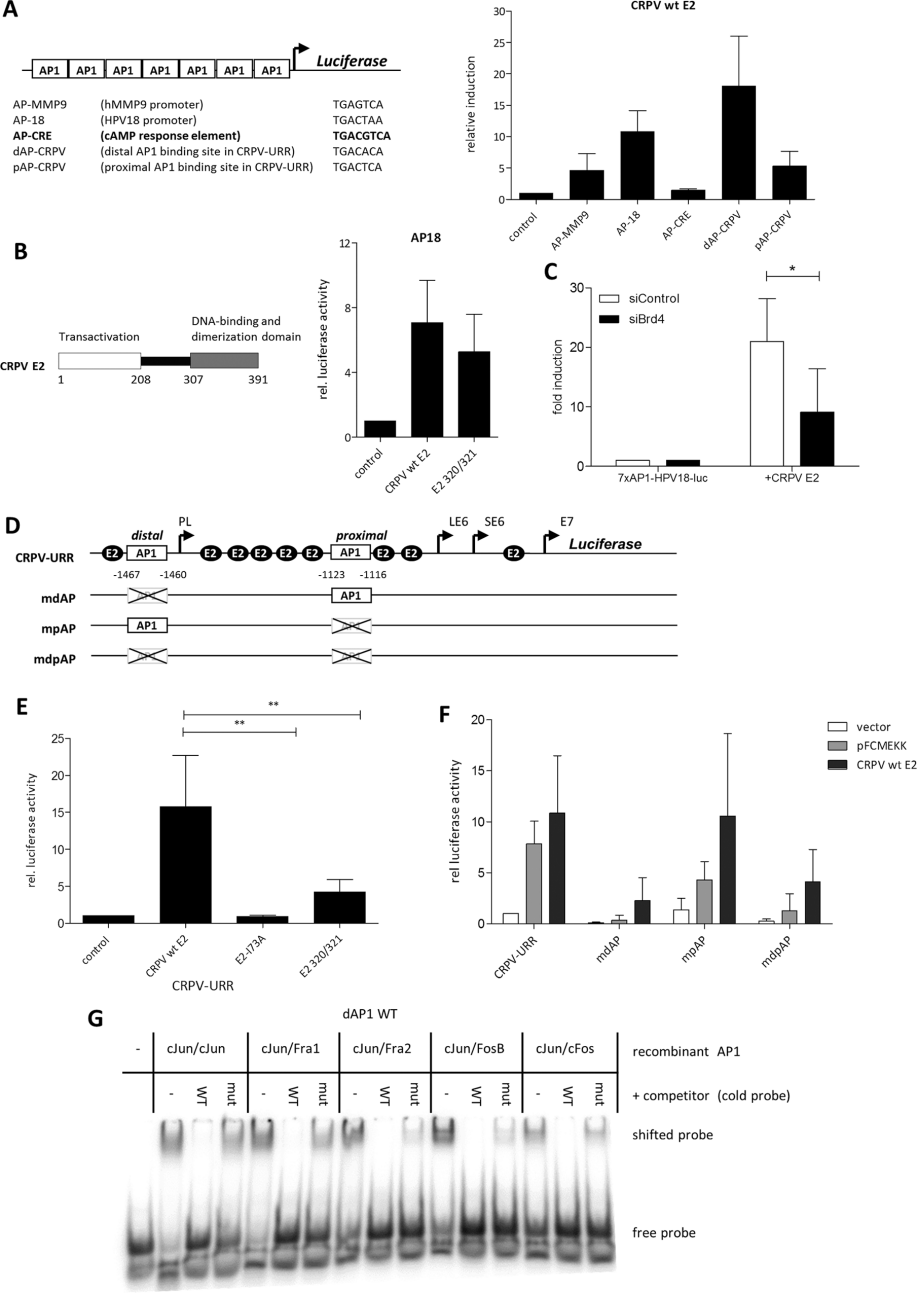
E2 transactivation of artificial AP1-dependent promoters and of the natural CRPV enhancer/promoter (A) Left panel: Schematic overview of the artificial AP1/CRE dependent reporters. Right panel: Relative activation in C33A cotransfected with wt E2 or empty vector as a control (which was set to 1) and the different AP1-dependent reporters (B) Left panel: schematic illustration of CRPV E2. Right panel: Activation of the multimerized HPV18 AP1BS by wt E2 and DBD mutated E2 (CRPV E2 K320M/C321R) relative to the empty vector control. (C) Activation of the 7xAP1-HPV18-luc by CRPV E2 after Brd4 silencing. C33A cells (~7x10^4^) were transfected with 150ng Brd4 siRNA (siBrd4-1) or a control siRNA (siControl). 24h later the cells were transiently transfected with 50ng of the reporter (7xAP1-HPV18-luc) and 5ng of the E2 expression vector or the empty vector (pSG). Serum starvation of the cells started 5h after DNA transfection. Measurement was carried out 48h after DNA transfection. The induction of the 7xAP1-HPV18-luc by E2 is shown as fold induction relative to the reporter transfected with the empty expression vector (= 1). The values indicate mean ± SEM of four independent experiments. Paired one tailed t test was used for statistical significance (*p<0.05). (D) Schematic description of the wt CRPV URR reporter and the reporters mutated in AP1BS used in the luciferase assays. Distal and proximal (dAP-CRPV and pAP-CRPV) indicates the position of the AP1BS relative to the CRPV E6 promoter (E) CRPV-URR reporter induction by wt E2, CRPV E2 K320M/C321R (E2 320/321) and E2-I73A proteins. The empty vector (control) was used as a control and set to 1. Unpaired two tailed t test was used for statistical significance (**p<0.01). (F) Activation of the CRPV-URR reporters shown in Fig 1D, by wt E2 and pFCMEKK (MAP3K1) as a positive control for AP1-dependent activity. (G) EMSA with purified recombinant AP1 complexes and ^32^P-labelled ds oligo matching the distal AP1BS (WT) in Fig 1D and unlabeled wt as well as mutant ds oligo as competitor (cold probe).

To study the ability of CRPV E2 to activate AP1-dependent reporters, we performed luciferase experiments with artificial promoter constructs consisting of multimers of four different TPA- (TREs) and as control one cAMP-responsive element (CRE). Seven copies of each sequence element originating from the human MMP9 promoter (AP-MMP9), from the URR of HPV18 (AP18), from the URR of CRPV (dAP-CRPV, pAP-CRPV) and from the CRE element were cloned upstream of the luciferase gene (Fig 1A) into the vector pLuc-MCS (Stratagene Corp. La Jolla, CA) and co-transfected with wild-type (wt) CRPV E2 in HPV-negative cervical carcinoma C33A cells (ATCC). Wt CRPV E2 activated all TRE-dependent reporters (5- to 18-fold), but not the CRE reporter (Fig 1A). As no E2BSs are present in the investigated promoter regions, the E2-mediated stimulation of the AP1 reporters seems to occur without specific binding of E2 to the reporter plasmids.

### The DNA-binding domain of CRPV E2 is dispensable but interaction with Brd4 is essential for AP1 activation

Our previous studies demonstrated that the AP1-mediated activation of the MMP9 promoter by CRPV E2 does not require E2BS in the promoter region [13]. Therefore we tested if the CRPV E2 DNA-binding domain (DBD) is dispensable for activation of an AP1 reporter. Using a DNA-binding deficient E2 mutant with two amino acid substitutions in the highly conserved DNA binding domain (DBD; CRPV E2 K320M/C321R) [5,13], we found a complete loss of the ability to activate an E2-dependent reporter construct (pC18-Sp1-Luc) and to cooperate with E1 in the replication of the CRPV-URR (S1A Fig). Nevertheless, co-transfection of CRPV E2 K320M/C321R with AP18 into C33A cells resulted in activation of AP18 comparable to the wt CRPV E2 (Fig 1B), suggesting that the DBD of E2 is not required for the activation of AP1 elements.

Since Brd4 interacting with PV E2 proteins is required for stability of E2 and its transactivating capability [6,8], we tested whether Brd4 is also necessary for AP1 activation. Specific knockdown of Brd4 was performed with an siRNA directed against Brd4 that was co-transfected with CRPVE2 and the AP18 reporter construct into C33A cells. Four independent experiments demonstrated a clear reduction of the E2-mediated induction of the AP18 reporter in the presence of the siRNA against Brd4 (Fig 1C). In addition, co-transfection studies were performed with a dominant negative inhibitor form of Brd4 (pcDNA4C-SV40NLS-hBrd4-CTD) [21] that was previously reported to inhibit E2-mediated activation of E2BS-dependent promoters [21–23]. pcDNA4C-SV40NLS-hBrd4-CTD was transfected in increasing amounts together with a constant amount of CRPV E2 and the AP18 reporter construct into C33A cells. As a result, a dose-dependent decrease of the activation of the AP18 reporter devoid of E2BS by E2 was observed (S1B Fig).

### The natural enhancer/promoter in the URR of CRPV is dependent on AP1

To overcome limitations using artificial reporters with multimerized AP1BS, we next investigated the natural CRPV URR containing nine E2BS and two putative AP1BS. The entire URR encompassing the L1 stop codon and the E7 ATG driving the luciferase reporter was cloned (CRPV-URR; Fig 1D) and cotransfected with wt CRPV E2 or CRPV E2 proteins mutated in either the DBD (K320M/C321R) or the Brd4-binding domain (I73A) (Fig 1E). The E2 DBD mutant still caused activation (4-fold) compared to wt E2 (15-fold), whereas the Brd4-binding-deficient E2 mutant (I73A) completely lost the ability to transactivate the URR (Fig 1E). The ability of the E2 DBD mutant to still activate might be explained by the presence of AP1BS in the natural CRPV URR. To investigate this in more detail, we mutated either one or both of the putative AP1 elements and co-transfected the resulting luciferase reporter constructs with CRPV E2. As positive control we used an expression vector for the MAP3K1 protein (pFC-MEKK, Stratagene, La Jolla, CA,) [24] known to generally activate the MAPK signal transduction pathway. The induction of the CRPV-URR by MAP3K1 expression (7-fold) indicates that the viral enhancer/promoter is responsible to stimuli acting via AP1 (Fig 1F). Mutation of the single distal or both distal and proximal AP1BS affected the basal activity of the reporter construct and abolished the inducibility via MAP3K1, whereas mutation of the proximal AP1BS (mpAP) had a less profound effect. Interestingly, activation of the CRPV-URR by E2 was strongly affected by the mutation of the single distal or both AP1BS despite the presence of nine intact E2BS, while mutation of the proximal mpAP did not change the extent of transactivation by E2 (Fig 1F).These data suggest that AP1BS play a major role for the activity of the CRPV-URR both in the absence and the presence of E2 and that the distal AP1 site, which is a canonical AP1 site (TGACACA) is the dominant one. Gel mobility shift experiments using a DNA oligonucleotide carrying the sequence of the distal AP1 site and different recombinant AP1 dimers (cJun/cJun; cJun/Fra1; cJun/Fra2; cJun/FosB; cJun/cFos) co-expressed and purified from *E*.*coli* [16] demonstrated specific binding of those AP1 dimers to the distal AP1BS of CRPV, whereas binding to the mutated site (mdAP) was abolished (Fig 1G).

### AP1BS in the CRPV URR are required for CRPV tumorigenesis

**Fig 2:**
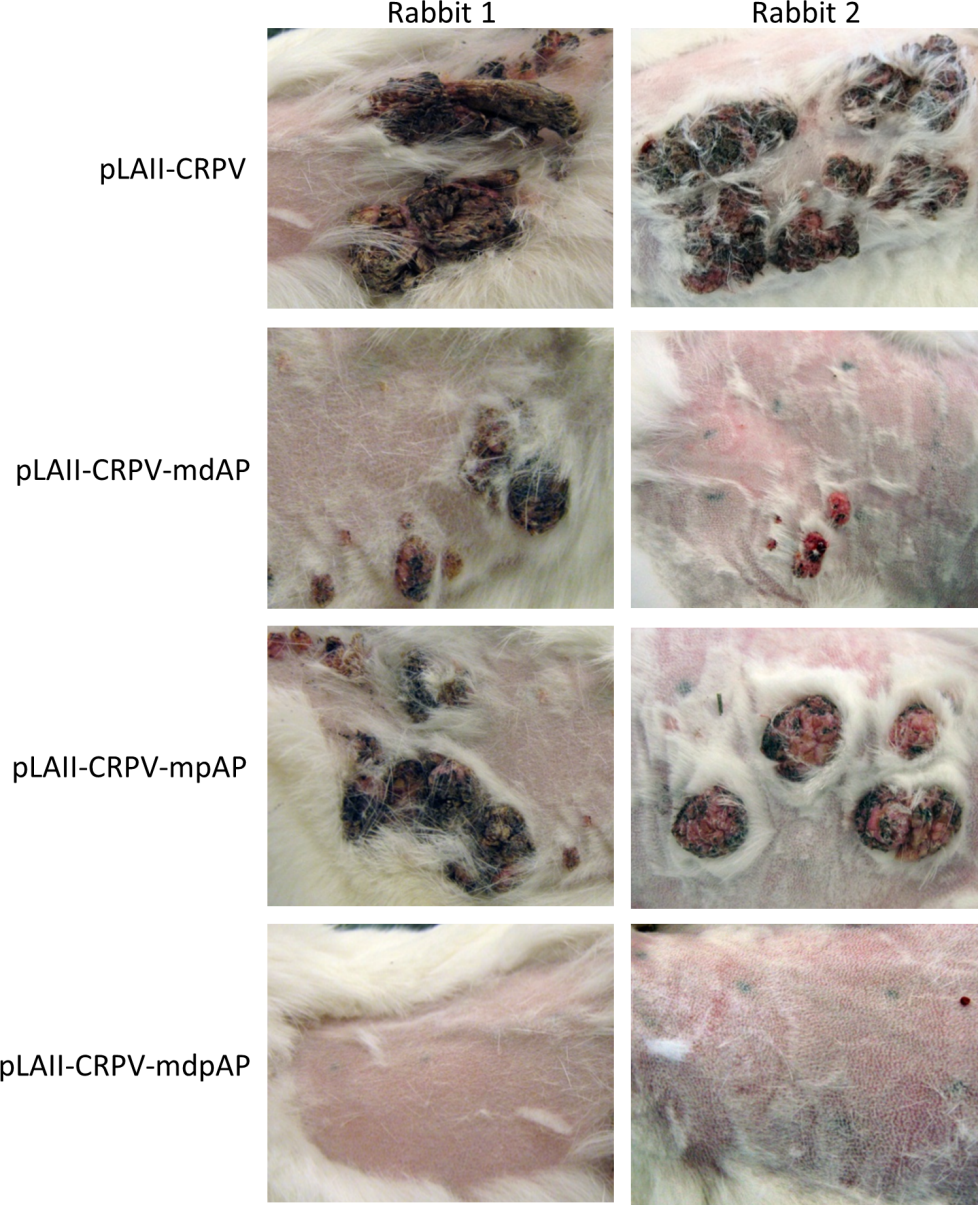
AP1BS in the CRPV-URR are required for tumor formation. Skin sites at the back of five rabbits were infected with wt CRPV (pLAII-CRPV) or a genome mutated in one of the AP1BSs within the URRpLAII-CRPV-mdAP, pLAII-CRPV-mpAP, or in both (pLAII-CRPV-mdpAP). Six skin sites in every rabbit were infected with each construct and pictures were taken 6 months post infection.

To study the role of the AP1BS for tumor induction *in vivo*, we introduced single mutations (mdAP, mpAP) and the double-mutation (dpAP) into the URR of the whole CRPV genome (pLAII-CRPV-mdAP, pLAII-CRPV-mpAP, pLAII-CRPV-mdpAP). The skin of New Zealand White (NZW) rabbits was infected with these CRPV genomes and tumor growth was assessed 6 months post infection. We observed only 33.3% papilloma induction with pLAII-CRPV-mdAP as compared to pLAII-CRPV-mpAP and the wt CRPV positive control (70% and 100%, respectively; Fig 2; Table 1A). No tumors were observed in animals infected with a CRPV genome mutated in both AP1 sites despite the presence of intact E2BS (pLAII-CRPV-mdpAP) (Fig 2 and Table 1A), demonstrating an essential role for AP1 in PV-induced tumorigenesis.

**Table 1:** Papilloma growth on infected rabbit skin 6 months post infection.

Construct	Total number of rabbits	Total number of infected skin sites	% papilloma induction
**A. Single and double mutation of AP1BS within the CRPV URR**			
pLAII-CRPV	5	30	100
pLAII-CRPV-mdAP	5	30	33.3
pLAII-CRPV-mpAP1	5	30	70
pLAII-CRPV-mdpAP1	5	30	0
**B. Brd4 knockdown**			
pLAII-CRPVshLuciferase	3	18	100
pLAII-CRPVshBrd4-1	3	18	0
pLAII-CRPVshBrd4-2	3	18	67
**C. MEK1 knockdown**			
pLAII-CRPVshLuciferase	2	12	100
pLAII-CRPVshMEK1-1	2	12	8
pLAII-CRPVshMEK1-2	2	12	42

### Brd4 is essential for CRPV to induce tumors

**Fig 3:**
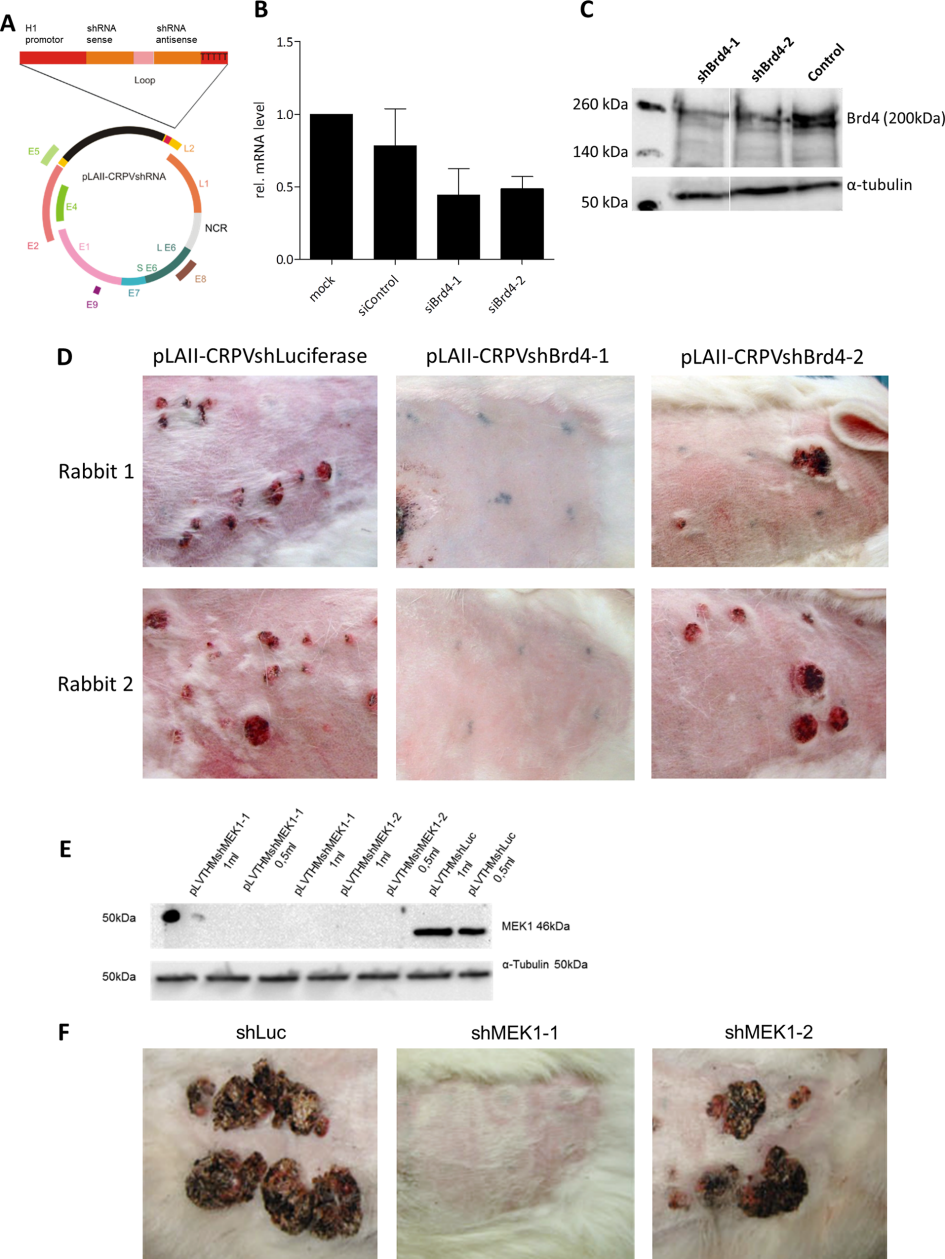
In vivo knockdown of Brd4 or MEK1 reduces papilloma growth in rabbits. (A) Schematic view of the recombinant CRPV genome carrying the shRNA expression cassette (pLAII-CRPVshRNA). (B) Brd4 silencing by three different siRNAs 48h after transfection into AVS cells [27]. siControl: Allstar; Negative Control siRNA; Mock: HiPerFect reagent. (C) Brd4 silencing in 293T cells transduced with lentiviral vectors carrying different shRNAs (pLVTHMshBrd4-1/-2); negative control pLVTHMshLuciferase. Alpha-tubulin was used as loading control. Molecular weight is indicated on the left. (D) Rabbit skin infected with pLAII-CRPVshRNA directed against Brd4 and with pLAII-CRPVshLuciferase. Six skin sites were infected with each construct per rabbit. The injection sites are marked with tattoo ink. Images were taken 6 months post infection. (E) MEK1 silencing in AVS cells transduced with lentiviral vectors carrying different shRNAs (pLVTHMshMEK1-1/-2); negative control pLVTHMshLuciferase. Alpha-tubulin was used as loading control. (F) Rabbit skin infected with pLAII-CRPVshRNAs directed against MEK1 and with pLAII-CRPVshLuciferase. Six skin sites were infected with each construct per rabbit. The injection sites are marked with tattoo ink. Images were taken 6 months post infection.

We previously observed that mutations of conserved amino acids within the E2 transactivation domain (TAD) that mediate Brd4 binding severely impaired the ability of CRPV to induce tumors in rabbits [2]. In the meantime we developed a recombinant CRPV genome harboring an shRNA cassette instead of the late L2 gene (pLAII-CRPVsh) [25], which allows the efficient knockdown of endogenous cellular proteins (Fig 3A). By using this construct, we directly assessed the significance of Brd4 for CRPV-dependent tumorigenesis in vivo. First, two different siRNAs against Brd4 (siBrd4-1, siBrd4-2) were transfected in rabbit keratinocytes immortalized with the whole CRPV genome [26]. Both siRNAs efficiently reduced Brd4 mRNA levels to 44% or 58% as compared to the mock-transfected control (Fig 3B). Using the identical sequences, shRNA expression vectors were constructed (pLVTHMshBrd4-1 and pLVTHMshBrd4-2) and transduced into 293T cells (ATCC) where a reduced Brd4 protein level in comparison to the control shRNA was detected (Fig 3C). The shBrd4-1/2 sequences were then cloned into the shRNA expression cassette of pLAII-CRPVsh (Fig 3A) and NZW rabbits were infected with the resulting constructs. As negative control, an shRNA sequence targeting the firefly luciferase gene was used (pLAII-CRPVshLuc). Six months post infection no tumors were observed in pLAII-CRPVshBrd4-1 infected rabbits (Fig 3D and Table 1B), while pLAII-CRPVshBrd4-2 diminished tumor induction to 67% as compared to the control (pLAII-CRPVshLuc; Table 1B). These results strongly support an important role of Brd4 for CRPV-mediated tumor formation *in vivo*.

### Gene expression profiling in C33A cells inducible for E2 identified c-Fos and FosB as targets regulated by E2 proteins of different HPV types

**Fig 4:**
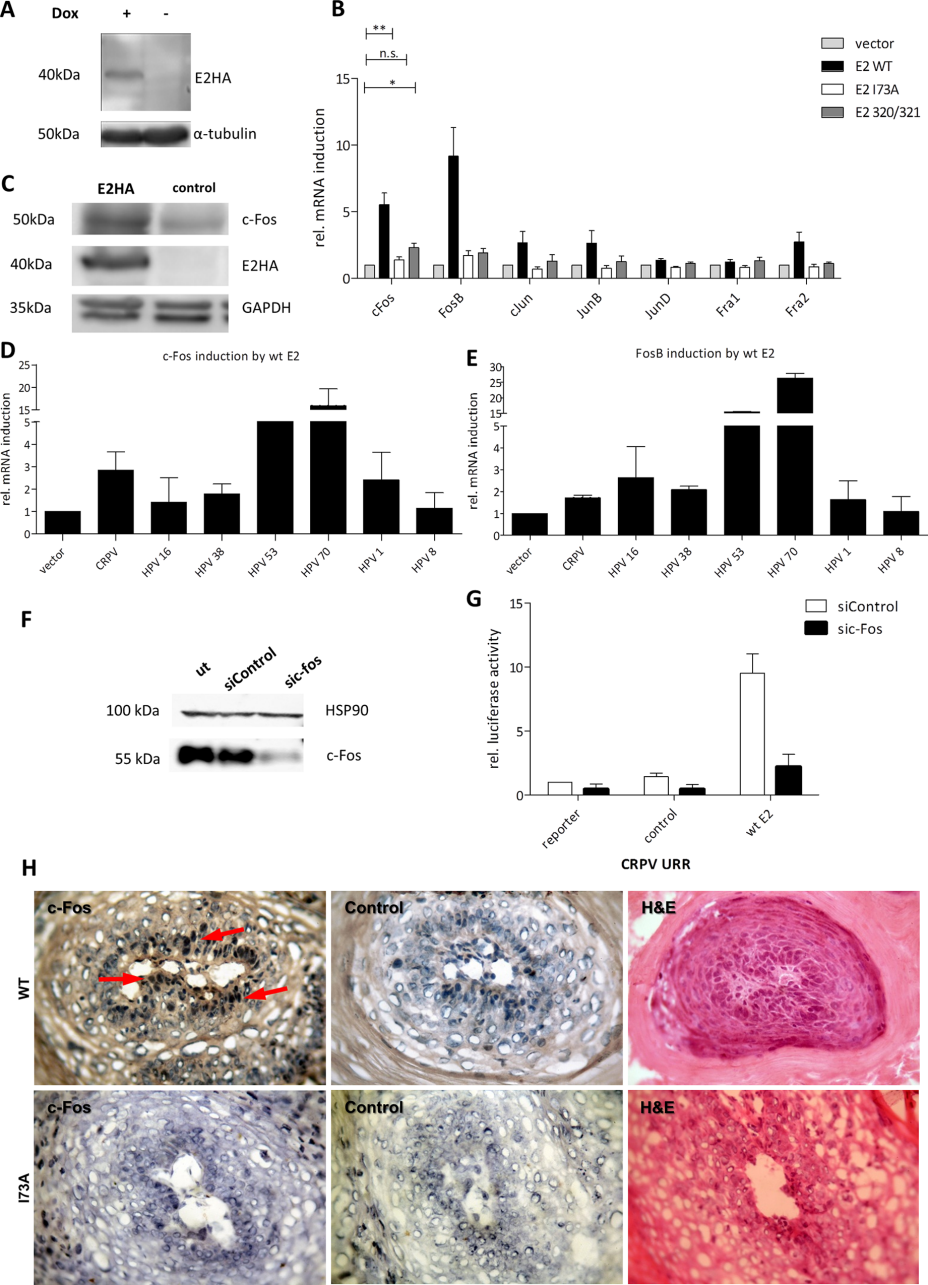
The cellular gene *c-Fos* inducible by E2 is a component of the AP1 complex driving the viral promoter. (A) Western blot of the inducible C33A cell line for detection of HA-tagged E2 48h after addition of doxycycline (+). Alpha-tubulin was used as a loading control. (B) C33A cells were transfected with empty vector (control) and either wt E2, E2-I73A or K320M/C321R (E2 320/321). 48 h later the cells were harvested and analyzed for AP1-monomers mRNA induction. Unpaired two tailed t test was used for statistical significance (*p<0.05, **p<0.01). (C) Western blot analysis of E2 and c-Fos expression compared to the empty vector (control). C33A cells were transfected as before and treated with 10 μM lactacystin 6h before harvest to prevent protein degradation. GAPDH was used as a loading control. (D) c-Fos and (E) FosB mRNA induction by E2 proteins from different PVs. C33A cells were transfected as before and analyzed for c-Fos and FosB mRNA induction compared to the control (empty vector). (F) Western blot of C33A cells that were transfected with a pool of three c-Fos siRNAs (sic-Fos) and a control siRNA (siControl). 10 μM of MG-132 was added to the medium 6h before harvest. HSP90 was used as a loading control. (G) Activation of the wt CRPV-URR by wt E2 after c-Fos silencing. The values are related to the basal activity of the CRPV-URR reporter co-transfected with empty vector and the siControl. (H) Immunohistochemistry of I73A and wt CRPV induced papillomas using an antibody against c-Fos. Control: Incubation with secondary antibody only. The arrow indicates nuclear c-Fos staining.

To identify the mechanism of E2-mediated activation of AP1-responsive promoters, we used an unbiased approach to search for cellular genes with altered expression levels in the presence of CRPV E2. For this we established an inducible E2-expressing cell line using the pRTS1-vector [28]. The system prevents the expression of the target gene through a tetR-KRAB fusion protein, which is released after the addition of doxycycline, whereas the co-expressed tetR-VP16 protein simultaneously mediates activation. C33A cells harboring the pRTS1-CRPV E2-HA plasmid were induced for 48h and induction of E2 was verified at protein levels (Fig 4A). Total cellular RNA was extracted 48h after induction and analyzed by the Affymetrix GeneChip Human exon 1.0 ST Array. Gene expression analysis revealed 137 genes that were differentially expressed after induction of wt CRPV E2 (≥1.75-fold) (S1 Table). Surprisingly, c-Fos and FosB, two components of the AP1 family, were found to be transcriptionally upregulated. To validate this finding and extend this observation to other AP1 components, RNA was isolated from C33A cells transiently transfected with wt CRPV E2, CRPV E2 K320M/C321R, and the empty vector as control. qPCR confirmed the induction of c-Fos (6-fold) and FosB (8-fold) by wt CRPV E2 and by the DNA-binding-deficient E2 mutant (2-fold) (Fig 4B). The upregulation of c-Fos occurred also at the protein level (Fig 4C). To extend our findings to other papillomavirus types, we transiently transfected C33A cells with vectors expressing the E2 protein of different HPV types [29]. As a result we found that most E2 proteins were able to induce c-Fos as well as FosB in C33A cells (Fig 4D and 4E) and also in HPV18-positive HeLa cells (ATCC) (S2 Fig). Based on the evidence of c-Fos involvement in skin tumorigenesis [19,30], we decided to focus on c-Fos in the following experiments.

Hence, we tested whether c-Fos–as one possible component of the AP1 complex–contributes to the *in vitro* activity of the CRPV URR either in the presence or absence of CRPV E2. For this, c-Fos was silenced in C33A cells using a pool of three siRNAs (Fig 4F) before transfecting the reporter plasmid and then cells were serum starved prior to harvest. Silencing of c-Fos affected the basal activity of the CRPV-URR-Luc reporter, but not E2 expression levels (S3 Fig) and dramatically diminished E2-mediated activation as compared to the control siRNA (Fig 4G). This indicated that c-Fos was not only upregulated by E2, but is also part of the AP1-complex acting as a major stimulus on the promoter responsible for the expression of viral E6/E7 oncogenes. To study in vivo c-Fos regulation by wt CRPV genome, we characterized c-Fos expression by immunohistochemistry. We found up-regulation of c-Fos only in CRPV-induced papillomas in contrast to normal healthy rabbit skin (S4 Fig). In addition, we performed immunohistochemistry on sections derived from papillomas that occurred after infection with a CRPV genome containing a mutation at amino acid 73 (I73A) of the E2 protein. This mutation was shown to cause a replication-competent, but transactivation-deficient phenotype with a strongly reduced ability to induce papillomas that appeared at a much later time-point in comparison to the wt CRPV control infection [2]. The I73A mutation has been shown to disable E2 from binding to Brd4 [5]. Our data show that this causes a loss of the ability to induce c-Fos in vivo in comparison to the wt control (Fig 4H).

As it has been shown that mild irritation of the skin before infection with CRPV DNA greatly increases tumor formation in rabbits [31], we speculated that this might be due to the induction of the immediate early gene c-Fos. To test for this, punch biopsies were taken 10, 20 and 30 minutes after manual irritation of the skin with sand paper and cellular RNA was analyzed by qPCR. In comparison to untreated skin, c-Fos mRNA was rapidly induced in irritated skin up to 3-fold 30 minutes after irritation (S5 Fig), supporting an important role of c-Fos for the establishment of an infection with CRPV *in vivo*.

To generate in vivo data supporting the role of c-Fos in tumorigenesis, we could not use our recombinant shRNA-CRPV genome to directly knock down c-Fos, as we already had to use a pool of three different siRNAs for in vitro knockdown. We therefore developed two shRNAs for MEK-1 as an important regulator of c-Fos expression, which demonstrated in tissue culture a remarkable knockdown of the MEK-1 protein (Fig 3E). When those shRNAs were tested in vivo, they showed a dramatic effect on tumor induction (8% and 42%; Fig 3F and Table 1C), which supports a critical role of the MAPK pathway leading to the stimulation of c-Fos in tumorigenesis.

### Mechanism of c-Fos induction by E2

**Fig 5:**
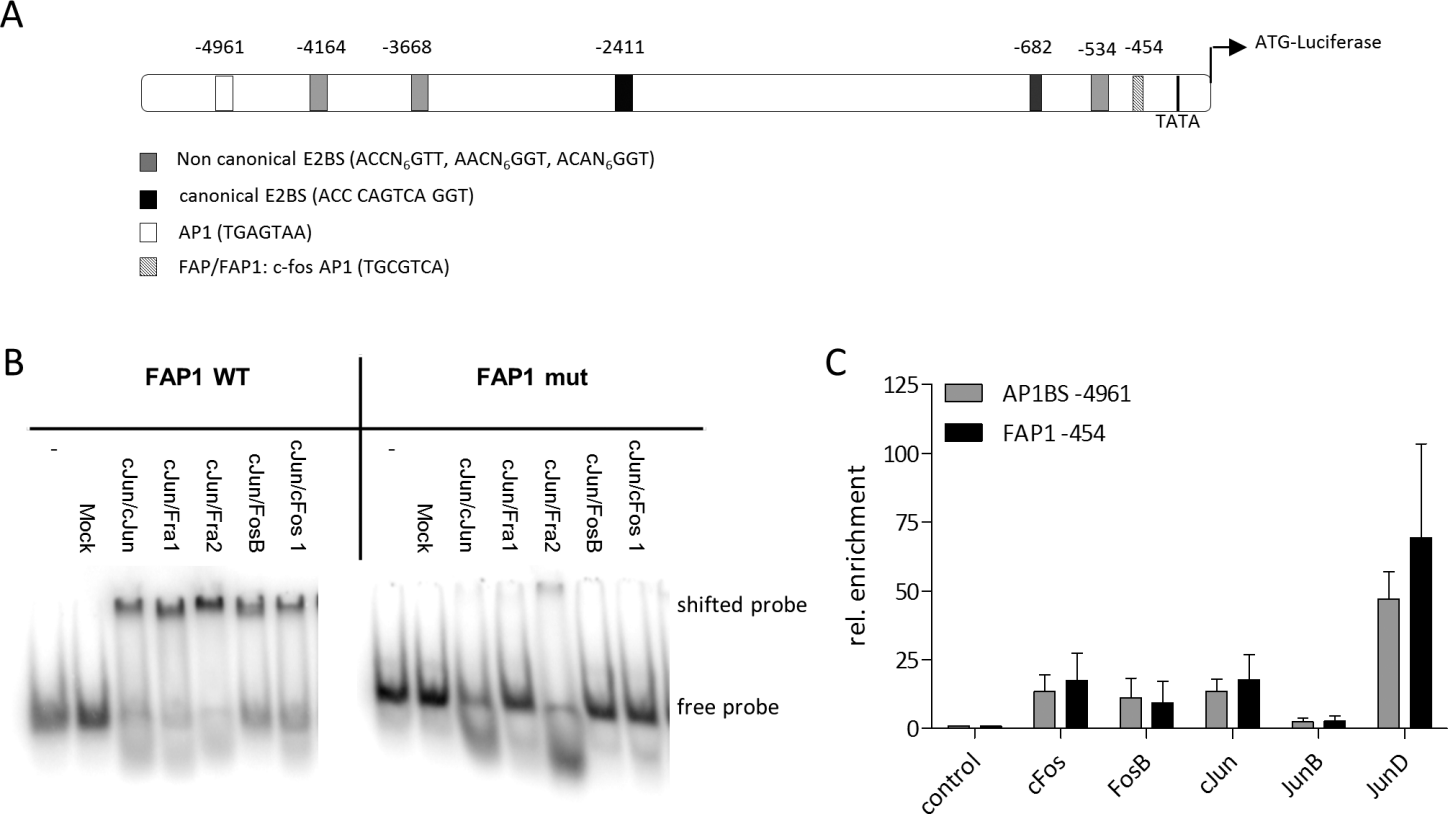
The c-Fos promoter is regulated via AP1BS. (A) Schematic description of the AP1 BS and E2BS in the region 5 kb upstream of the c-Fos ATG. Canonical and non-canonical E2BS are shown as black and white boxes, respectively. The FAP1 (FAP1) site is shown as hatched box, the AP1BS (AP1) as white boxes. (B) Electrophoretic mobility shift assay (EMSA) with purified recombinant AP1 complexes and ^32^P-labelled ds oligos matching the distal FAP1BS (FAP1 WT) and the mutant FAP1 sequence (FAP1 mut) in the c-Fos promoter. (C) ChIP analysis of C33A cells after serum starvation for 18h and induction with serum for 1h. Chromatin was isolated after crosslink with formaldehyde and precipitated using the indicated antibodies. Samples were analyzed by qPCR with specific primers flanking the AP1BS at -4756bp (grey bars), and the FAP1BS at -249bp (black bars).

First we performed luciferase reporter experiments with consecutively shortened c-Fos promoter fragments from -5238 till -362 bp in relation to the c-Fos ATG driving the luciferase gene (Fig 5A). The -2795 fragment had the highest activity and truncation of the -530 fragment to -362 caused further loss of activity (S6 Fig). In silico analysis of the nucleotide sequences revealed the presence of a canonical E2BS at position -2411 within the -2795 fragment and a c-Fos/AP1-site (FAP1 [32]) in the -530 fragment that is lost by truncation in the shorter -362 fragment [33]. Another potential AP1BS was detected at position -4961. When we mutated the FAP1 site within the -530 fragment, we observed a loss in activity similar to the truncated -362 fragment, which no longer contains the FAP1 site (S6 Fig). These results pointed towards an important role of the E2BS and FAP1 in the regulation of c-Fos activity. Therefore we first analyzed the binding of AP1 to FAP1 by gel-shift mobility experiments using a ds oligo matching either the wt or the mutated FAP1-sequence. Our results confirmed specific binding of different AP1 dimers (cJun/cJun; cJun/Fra1; cJun/Fra2; cJun/FosB; cJun/cFos) co-expressed and purified from *E*.*coli* [16] to FAP1, which is completely abolished with the mutated mFAP1 (Fig 5B).

We next performed chromatin immunoprecipitation (ChIP) experiments and investigated the binding of different AP1 components to FAP1 and the potential AP1BS at -4961 in the endogenous c-Fos promoter. C33A cells were serum-starved for 18h and after addition of serum ChIP experiments were performed using antibodies targeting respective AP1components c-Fos, FosB, c-Jun, JunB and JunD and primers flanking the AP1BS at positions -454 and -4961. All AP1 members, except JunB, were enriched at the FAP1 site and at the AP1 site at position -4961 (Fig 5C).

**Fig 6:**
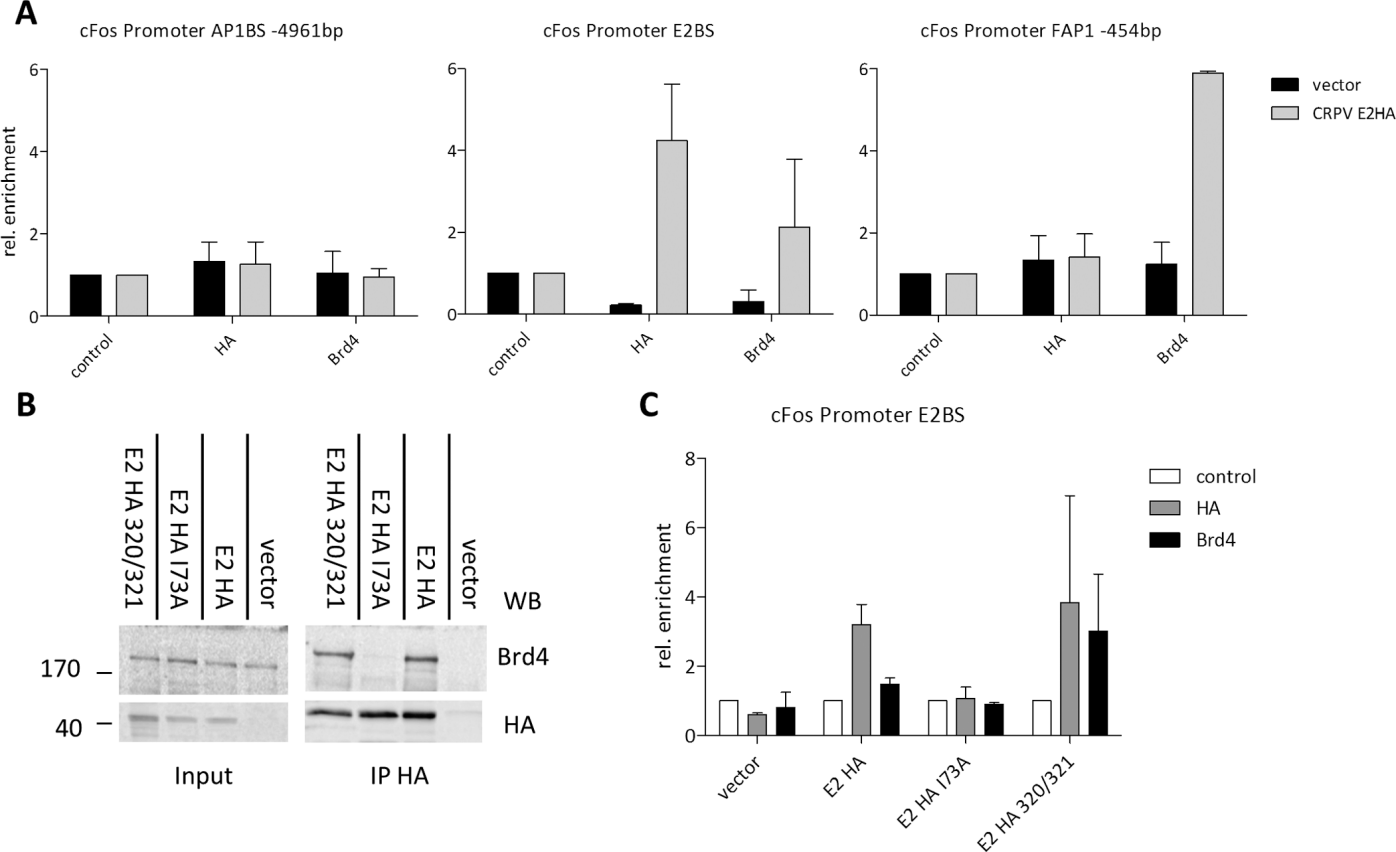
The c-Fos promoter is regulated via E2, AP1 and Brd4. (A) ChIP analysis of three independent experiments using C33A cells transiently transfected with CRPV E2HA (grey bars) and the empty vector (control; black bars). Specific primers flanking the AP1BS at -4961 (AP1BS -4961), the canonical E2BS at position -2411 (E2BS) and the FAP1 at -454 (FAP1–454) were used to analyze respective ChIP samples. (B) Co-IP of C33A cells stably expressing CRPV E2HA, CRPV E2HA I73A, CRPV E2HA K320M/C321R and the empty vector were treated with MG132 for 16h to prevent protein degradation. Samples were precipitated using an anti HA or anti-Brd4 antibody and analyzed by Western blotting for HA and Brd4. Molecular size in kDa is indicated on the left. (C) ChIP analysis of two independent experiments. C33A cells were transiently transfected with CRPV E2HA, CRPV E2HA I73A, CRPV E2HA K320M/C321R and the empty vector and crosslinked chromatin was immunoprecipitated with HA and Brd4 antibodies. In the control sample no antibody was used. Precipitated DNA was analyzed using specific primers flanking the canonical E2BS at position -2411.

To investigate how E2 activates c-Fos expression, binding of E2 and Brd4 to the consensus E2BS in the c-Fos promoter at position -2411 as well as to both AP1 sites was investigated by ChIP. A distinct enrichment compared to the empty vector of both CRPV E2-HA (4.1 fold) and Brd4 (2-fold) at the canonical E2BS was observed, indicating that CRPV E2 indeed binds together with Brd4 to this sequence. In contrast, no enrichment of E2 or Brd4 was observed at the AP1BS at -4961 while Brd4, but not E2, was enriched approximately 6-fold at the FAP1 (Fig 6A). This might be due to the close proximity of FAP1 to the transcription start site and the general transcriptional co-activator activity of Brd4 [3].

To address the binding requirements for E2 at the canonical E2BS in the c-Fos promoter, we first confirmed the E2-Brd4 interaction in our C33A cells stably expressing CRPV E2HA, CRPV E2HA I73A and CRPV E2HA K320M/C321R in co-immunoprecipitation experiments. Due to the extremely low expression levels, cells were pretreated with MG132 to detect E2 protein. As expected, wt CRPV E2HA and CRPV E2HA K320M/C321R interacted with endogeneous Brd4, whereas CRPV E2HA I73A did not (Fig 6B). In order to yield a higher protein amount of wt CRPV E2, CRPV E2HA I73A and CRPV E2HA K320M/C321R for ChIP experiments, approximately 8x10^7^ C33A cells were transiently transfected with the respective expression vectors for ChIP experiments. Again a specific enrichment of both CRPV E2-HA and Brd4 at the canonical E2BS was observed (Fig 6C). In contrast, the I73A mutant showed no enrichment, possibly due to a lack in protein stability caused by the inability to bind to Brd4 [2,34]. The E2K320R/C321M mutant displayed a comparable enrichment as shown for wt E2 at the E2BS of the c-Fos promoter, which correlates with the binding of Brd4. This is in line with our results showing a weak c-Fos induction at the mRNA level by the DNA-binding-deficient CRPV E2 mutant K320R/C321M (Fig 4B). Our data strongly support that CRPV E2 together with Brd4 activates c-Fos transcription by binding in complex to the E2BS in the c-Fos promoter.

## Discussion

We here report a novel mechanism how the complex of E2/Brd4 is able to activate the papillomavirus early promoter responsible for expression of the viral oncogenes via AP1 sites that are bound by dimeric AP1 complexes which contain c-Fos. Further we show that the presence of intact AP1 sites in the CRPV genome as well as of the E2 binding partner Brd4 is essential for tumor formation in the rabbit.

These observations explain earlier findings that AP1 is the major cellular transcription factor for the activity of the URR and that E2 requires additional cellular factors such as AP1, Sp1 and Oct1 for the ability to trans-activate [35,36]. We here show that E2 itself causes upregulation of c-Fos as one component of AP1, which is involved in the cell type specificity of papillomaviruses and the differentiation-dependent expression of viral genes in the differentiating epithelium [37,38]. Our data indicate that E2 transactivates the natural enhancer/promoter of PVs via stimulation of conserved AP1 binding sites in addition to binding to E2BS which are also required for the partitioning of the viral genome during mitosis of infected cells [5].

In addition, we demonstrate that any interruption of the E2-mediated transcriptional induction of the viral promoter through c-Fos either by mutating the AP1 binding sites in the genome or by knockdown of Brd4 affects the tumorigenic potential of papillomaviruses, although a possible side effect of both shRNAs used for knock down of Brd4 on other cellular oncogenes cannot be excluded. In the majority of cervical cancers caused by high-risk genital types, including HPV16, the E2 protein is lost because of the integration of the viral genome into the host chromosome. However, in those cases the viral genome integrates preferably in transcriptionally active genomic regions of the host and therefore the viral promoter underlies other transcriptional regulation mechanisms [39]. In cases of skin cancer or cervical cancer with mixed viral genome status or episomal DNA [40,41], E2 together with Brd4 might switch from a repressive effect on the viral promoter on integrated genomes to an activating effect on the episomal genome as has been proposed before [42,43] with AP1 playing a prominent role. In the case of high-risk genital type HPV31, the URR with several AP1 sites was in fact previously found to be induced by E2 in the absence of functional E2 binding sites [37,44] which might be achieved by stimulation of AP1 activity and subsequent activation of the viral promoter through the E2/c-Fos/AP1 pathway. This might also explain some of the earlier reports showing that low levels of E2 stimulate the viral promoter of, e.g., HPV18 [45], while higher levels lead to repression through the promoter-proximal E2BS.

In skin cancers caused by epidermodysplasia verruciformis PV types, which usually contain episomal viral genomes without repressive E2BS in the proximity of the early viral promoter, E2-mediated c-Fos induction could play a major role in tumorigenesis. This is supported by the finding that the E2 protein of the EV-papillomavirus HPV8 itself is able to induce skin tumors in transgenic mice [46], which indicates that the tumorigenic potential of E2 could be related to its ability to induce c-Fos. Interestingly, HPV-positive cells undergoing tumorigenic transformation experience a shift in the composition of the AP1 heterodimers [17,18]. Protein levels of c-Fos increased along with increasing tumorigenicity and a shift in AP1 complex composition from c-Jun/Fra-1 to c-Jun/c-Fos heterodimers was only observed in tumorigenic cells [17]. Others showed high expression of JunD and c-Fos in HPV-positive tumors, with close to no Fra-1 expression [47,48]. Interestingly, in this study we observed induction of c-Fos and Fos-B, but not Fra-1 by E2, which underlines a possible role of E2 in tumorigenesis. Furthermore it has been shown that c-Fos plays a major role in skin tumorigenesis. c-Fos-knockout mice that overexpressed a v-H-ras transgene developed papillomas that failed to undergo malignant conversion [19]. More recent data suggest that the suppression of squamous cell carcinoma is due to pharmacological inhibition of Fos/AP1 and p53/TACE reactivation [20]. While we have observed that E2 of CRPV and other HPV types induces c-Fos and Fos-B expression, we did not observe activation of Jun family members. However, when we quantified the expression levels of different Jun and Fos family members in C33A cells, we found rather high basal levels of c-Jun and JunD that would allow the formation of Jun/c-Fos dimers in C33A cells expressing E2 (S7B Fig). Interestingly, the human Fos-B promoter that we found to be activated by E2 also contains a canonical E2BS approximately -3500 bp of the transcription start site (TSS) as well as two non-canonical E2BS about 1kb upstream of the TSS, and the rabbit c-Fos promoter contains four identical non-canonical E2BS within 3kb upstream of the TSS. We did observe Brd4-dependent binding of CRPV E2 to a canonical E2BS (ACCCAGTCAGGT) with a spacer region containing 50% A/T nucleotides located at -2411 upstream of the ATG of human c-Fos. E2 did not bind to the FAP1 site closest to the transcription start site (-249) of c-Fos although we observed a clear enrichment of Brd4 at this site. Furthermore, the Brd4-binding-deficient E2 mutant I73A was not enriched at the canonical E2BS in the c-Fos promoter, which is probably due to a reduced stability of this mutant in the absence of Brd4 binding [5]. However, we observed an enrichment of the DNA–binding-deficient mutant E2 K320M/C321R together with Brd4 at the E2BS of the c-Fos promoter. This cannot be explained by binding of E2 in a sequence-specific manner as this mutant neither supports E2BS-dependent transcriptional activation nor replication in cooperation with E1. One possible explanation might be the binding of DNA by the N-terminal domain of E2 as described for BPV1 E2, which is supported by the presence of Brd4 at active cellular promoters [49,50]. Another possibility is that Brd4, which we show to be bound to the canonical E2BS, brings along E2 via its CTD to chromatinized DNA as shown for the hematopoetic transcription factor GATA-1 [51]. Notably we found Brd4 enriched at the FAP1 site in close proximity to the transcription start site exclusively in cells expressing E2 suggesting that only active promoters require the Brd4/P-TEFb complex for phosphorylation of the CTD of RNA polymerase II as shown for c-Fos driven by the cooperative action of E2 and Brd4 by binding to the canonical E2BS. The use of AP1 as a major driver of viral gene expression appears to be a general phenomenon as the viral promoters of HTLV1, KSHV and of JCV are also upregulated by AP1 [52–54]. Interestingly, other viruses also upregulate expression of c-Fos or Fos family members. Human T-cell leukemia virus type 1 (HTLV-1) directly upregulates c-Fos [55–57], while EBV [58] induces Fra-1/AP1 [59,60]. These observations support an important role of AP1 for the activity of tumor viruses. The ability of E2 to induce c-Fos was entirely dependent on Brd4, which recruits transcriptional regulatory complexes to acetylated chromatin and is a major interactor of all papillomavirus E2 proteins [6]. In-frame fusions of Brd4 with the NUT gene as observed in the aggressive NUT midline carcinoma demonstrated its role as an aberrant transcription factor that requires the bromodomains for its tumorigenic activity [61]. More recently, a direct, specific and acetylation-independent interaction of Brd4 with distinct transcription factors, such as p53, c-Jun and Myc/Max has been described [62]. Furthermore, the bromodomains of Brd4 also interact with specific acetylated regions of transcription factors, as has been shown for TWIST, a TF using Brd4 as co-activator controlling mesoderm formation during development [63].

The interaction of Brd4 with viral transcription or replication factors such as LANA from KSHV, Tax from HTLV1, large T antigen from MCPyV, EBNA1 and EBNA2 from EBV and E2 from PVs seems to be another conserved feature among tumor viruses and was shown to be responsible for viral promoter regulation as well as viral replication [64–67]. Because of its fundamental role in transcriptional regulation, Brd4 has also been investigated as a therapeutic target to combat a number of cancers with deregulated Brd4 activity [61]. The recent identification of Brd4 inhibitors including JQ1 and I-BET provides great potential for treatment of HPV-induced cancers as both inhibitors target bromodomains [68,69] and prevent them from binding to acetylated histones and to act as transcriptional activators [70].

In summary, we present evidence of a novel pathway in which the E2/Brd4 complex activates the papillomavirus promoter via c-Fos and we show that each of the three components is essential for tumorigenesis. Furthermore we demonstrate that E2 contributes in two different ways to tumorigenesis. First by stimulating the viral promoter responsible for the expression of the viral oncogenes via E2- and AP1-binding sites and secondly by stimulating c-Fos, which is involved in skin tumorigenesis independently of PVs. Because both E2-mediated regulation of viral oncogene and c-Fos expression are completely dependent on Brd4, our study supports the idea that bromodomain inhibitors as well as inhibitors of the MAPK pathway affecting protein levels of cellular AP1 may also be effective against PV-induced tumors, which requires further investigation. MAPK pathway inhibitors are supported by a recent study where BRAF inhibitors, such as vemurafenib, caused the appearance of ß-papillomavirus associated squamous cell carcinoma (SCC) in up to 26% of treated melanoma patients, while the combination of vemurafenib with the MEK-inhibitor cobimetinib reduced the appearance of SCC to 5% [71]. The identification of AP-1, E2 and Brd4 as crucial regulators for CRPV and cellular c-Fos and MMP9 promoter activity further substantiates their implications in regulating HPV gene expression and the interplay between viral and cellular factors modulating eukaryotic transcription [72].

## Materials and Methods

Construction of plasmids, cell culture conditions, transient luciferase assays, siRNA transfections, quantitative real-time PCR, lentiviral infection, microarray processing and data analysis, immunoblot analyses and detailed chromatin immunoprecipitation (ChIP) are listed in supplemental experimental procedures (S1 Text).

### Ethics statement

Animal experiments were reviewed and approved (Permit Number: H1/03 and H1/08) by the responsible authority (Regierungspräsidium Tübingen, Baden-Württemberg, Germany) according to the German Animal Welfare Act (TierSchG §8 Abs. 1) and were performed according to national regulations (TierSchVersV).

### Animal experiments

New Zealand White rabbits were obtained from Charles River Laboratories. Rabbits were infected with different recombinant CRPV genomes using the “helios gene gun” (Bio-Rad) as described previously [2]. Tumor growth and papilloma size were regularly monitored and documented. Skin punches (6mm in diameter) from normal rabbit skin were taken using sterile single use Biopsy punches (pfmmedical) and total RNA was extracted using Qiazol (Qiagen) followed by purification of the RNA with RNeasyMinElute columns (Qiagen) according to the manufacturer´s instructions.

### Generation of doxycycline-inducible C33A/CRPVE2-HA cell lines

C33A cells were transfected with pRTS1-CRPVE2-HA or empty vector pRTS1 and selected with 250 μg/ml of hygromycin for 5 days. CRPV E2-HA expression was induced in stable pooled cell lines by the addition of 1 μg/ml of doxycycline for 48h. The cells were then harvested and total cellular RNA was isolated for microarray analysis.

### Electrophoretic mobility-shift assay

Double-stranded oligos (sequences listed in S2 Table) were labelled with ^32^P (GE Healthcare) using Polynucleotide Kinase (Fermentas) and purified with illustra ProbeQuant G-50 Micro columns (GE Healthcare). 5–10 fmol/μl were used in binding reactions with 50 ng of recombinant protein in Bclw/BSA buffer (10% Glycerol, 10 mM Hepes pH 7.9, 70 mM NaCl, 0.2 mM EDTA, 4 mM MgCl_2_, 25 mM DTT, 50 μM Zn, 0.1 mg/ml BSA and 50 ng/μl polydI-dC). Binding reactions were performed for 30–40 min at 30°C and separated on a 4% polyacrylamide gel with 0.25x TBE (89 mM Tris, 89 mM boric acid, and 1 mM EDTA) as running buffer for 1h. The gel was dried, exposed and then visualized by using Typhoon 9200 PhosphorImager (GE Healthcare). For oligo competitions, a 100-fold molar excess of unlabeled DNA fragments containing either wild-type or mutated distal AP1 sequences was included at the beginning of the reaction.

### Co-immunoprecipitation (Co-IP)

C33A stably expressing CRPV E2HA, CRPV E2HA I73A, CRPV E2HA K320M/C321R or the empty vector (pIRESpuro3, Clontech) were seeded and treated with 1 μM MG132 16 h before harvest to prevent protein degradation. Cells were harvested in cold PBS, lysed in high salt NP-40 buffer (50 mM Tris-HCl, pH 8.0, 400 mM NaCl, 0.1% NP-40, and 0.25% sodium deoxycholate, 1 mM DTT and Protease-Inhibitors (Roche) and incubated for 30 min on ice. The lysate was centrifuged, and the supernatant was diluted to a final NaCl concentration of 150 mM. After that, the supernatant was incubated with 30 μl of anti HA-magnetic microbeads (Miltenyi biotec) for 1h. The supernatant was discarded and the beads were washed 2–3 times with NP-40 buffer containing 150 mM NaCl. Bound protein complexes were eluted using SDS sample buffer (Carl Roth).

### Chromatin Immunoprecipitation (ChIP)

For transfection for ChIP assays, one 100-mm plate per construct of C33A cells was transfected with 10 μg DNA using Fugene HD (Promega) according to the manufacturers’ instructions. ChIP was carried out as previously described [72]. See supplemental methods file for details.

### Statistical analysis

GraphPad (version 5) was used to calculate unpaired and paired p-values.

### Accession numbers

All transcriptional profiles have been submitted to the GEO database at NCBI (Accession no. GSE67345).

## Supporting Information

S1 Fig Wt and mutant E2 activities. (A) E2 K320M/C321R is unable to bind to DNA. Transient replication assay in C33A cells using the CRPV URR, CRPV E1, HA-tagged and untagged E2 and Brd4-deficient binding mutants of E2 (I73A, I73L and I73A HA-tagged (I73AHA)) as well with the DBD-deficient mutant E2 K320M/C321R (abbreviated to E2 320/321; untagged or HA-tagged). Wt E1 and E2 together with the empty vector were used as a control. (B) Brd4-CTD blocks activation of the AP18 reporter devoid of E2BS by E2 in a dose-dependent manner. Luciferase activity of C33A cells cotransfected with constant amounts of wt CRPV E2 and AP18 and increasing amounts of the dominant negative variant Brd4-CTD.
(TIF)

S2 Fig C-Fos mRNA induction in HeLa cells by E2 proteins from different HPVs. c-Fos mRNA induction by E2 proteins from different HPVs. HeLa cells (8x10^4^) were transiently transfected with 0.5 μg of empty vector (pSG) and wt E2 proteins. 48h later the cells were harvested and analyzed for c-Fos mRNA induction compared to the control (empty vector).
(TIF)

S3 Fig C-Fos silencing does not interfere with activation of the pC18-SP1-luc by CRPV E2. Activation of the pC18-SP1-luc by CRPV E2 after c-Fos silencing. C33A cells (~ 7x10^4^) were transfected with a pool of three c-Fos siRNAs (siFos) or a control siRNA (siControl). 24h later the cells were transiently transfected with 10ng of the reporter (pC18-SP1-luc) and 10ng of the E2 expression vector (CRPVE2) or the empty vector. Measurement was carried out 48h after DNA transfection. The induction of the pC18-SP1-luc is shown in relative light units (RLUs). The values indicate mean ± standard deviation of four independent experiments.
(TIF)

S4 Fig C-Fos is up-regulated in CRPV-induced papillomas. Immunohistochemistry of normal skin and of CRPV induced papillomas using an antibody against c-Fos. Control: Incubation with secondary antibody only. The dashed line indicates the basal lamina of the skin.
(TIF)

S5 Fig C-Fos mRNA induction in normal rabbit skin after light irritation with sand paper. The skin of 3 different rabbits was shaved and irritated using ultrafine sandpaper. Punch biopsies were taken after the indicated timepoints. C-Fos expression was analyzed by qRT-PCR and normalized to alpha-tubulin. Relative induction was compared to timepoint 0 min (before irritation). Standard deviation is indicated by error bars.
(TIF)

S6 Fig Truncation experiments of c-Fos reporter promoter constructs reveal the importance of a canonical E2BS for activation. Upper panel: Schematic description of the Δ c-Fos promoter reporters used in the luciferase assays. Canonical and non-canonical E2BS are shown as black and grey boxes, respectively. The FAP1 site is shown as hatched box, the AP1BS (AP1) as white box. (lower panel) Luciferase assay with truncated c-Fos-promoter constructs and wt E2 (grey bars) and mutant E2 constructs impaired in DNA- and Brd4-binding (E2-I73A-Gal4; black bars)) and the empty vector as control (dark grey bars). The induction of each construct is shown in relative light units (RLUs). The values indicate mean ±SD of five independent experiments.
(TIF)

S7 Fig Properties of the E2-inducible C33A cell line. (A) Protein expression levels of C33A cells transfected with HA-tagged CRPV E2 (overexpression) or empty vector (control). Alpha-tubulin was used as a loading control. Marker in kDa is indicated on the left. (B) cp-values of the mRNAs encoding different AP1 monomers in C33A cells transfected with CRPV E2 (wt E2) or empty vector (control).
(TIF)

S1 Table Differentially regulated genes as determined by microarray analysis. (XLSX)

S2 Table List of all primer sequences used in this manuscript. (PDF)

S3 Table List of all antibodies used in this manuscript. (PDF)

S1 Text Supplemental experimental procedures. (PDF)
