# Supplementary figures and images for: Papillomavirus-Associated Tumor Formation Critically Depends on c-Fos Expression Induced by Viral Protein E2 and Bromodomain Protein Brd4
Source: PLoS Pathog. 2016 Jan 4;12(1):e1005366. doi: 10.1371/journal.ppat.1005366 (PMC4699637; doi:10.1371/journal.ppat.1005366)

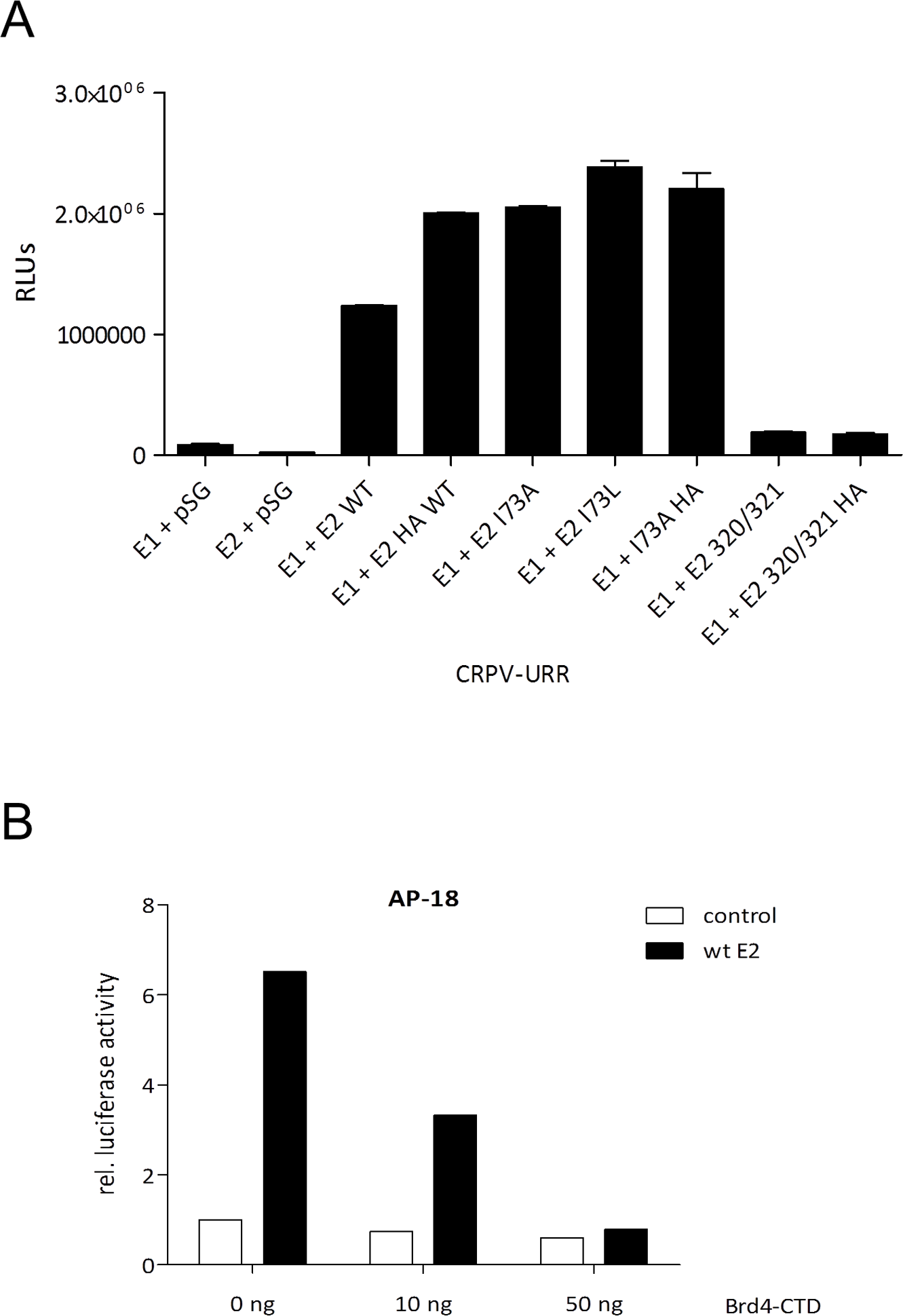

Supplement: S1 Fig — (A) E2 K320M/C321R is unable to bind to DNA. Transient replication assay in C33A cells using the CRPV URR, CRPV E1, HA-tagged and untagged E2 and Brd4-deficient binding mutants of E2 (I73A, I73L and I73A HA-tagged (I73AHA)) as well with the DBD-deficient mutant E2 K320M/C321R (abbreviated to E2 320/321; untagged or HA-tagged). Wt E1 and E2 together with the empty vector were used as a control. (B) Brd4-CTD blocks activation of the AP18 reporter devoid of E2BS by E2 in a dose-dependent manner. Luciferase activity of C33A cells cotransfected with constant amounts of wt CRPV E2 and AP18 and increasing amounts of the dominant negative variant Brd4-CTD. (TIF) [file ppat.1005366.s001.tif]

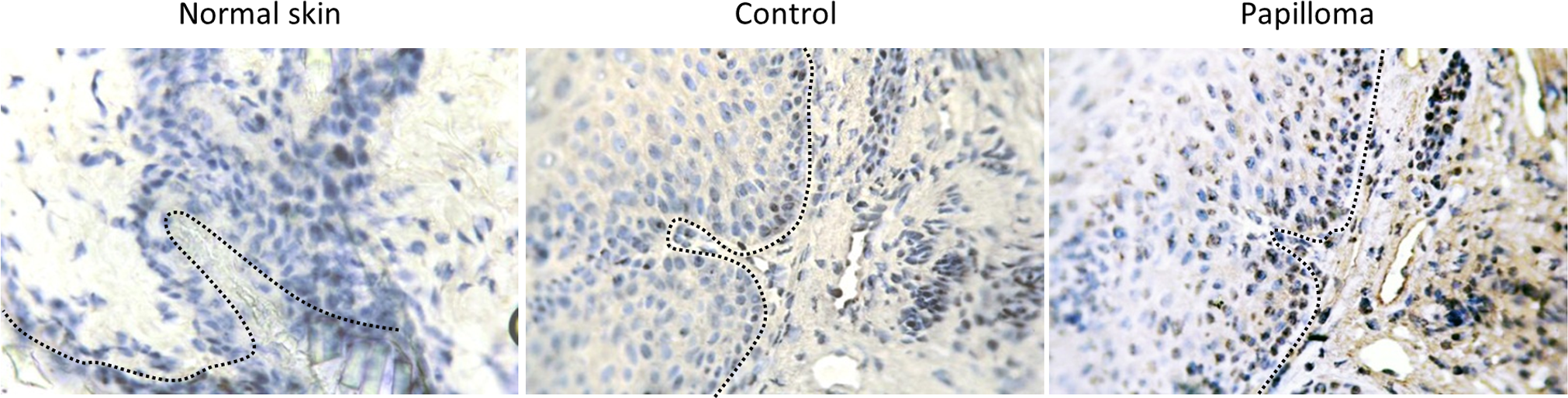

Supplement: S4 Fig — Immunohistochemistry of normal skin and of CRPV induced papillomas using an antibody against c-Fos. Control: Incubation with secondary antibody only. The dashed line indicates the basal lamina of the skin. (TIF) [file ppat.1005366.s004.tif]
