# Supplementary material for: Papillomavirus-Associated Tumor Formation Critically Depends on c-Fos Expression Induced by Viral Protein E2 and Bromodomain Protein Brd4
Source: PLoS Pathog. 2016 Jan 4;12(1):e1005366. doi: 10.1371/journal.ppat.1005366 (PMC4699637; doi:10.1371/journal.ppat.1005366)
Supplement: S2 Table — (PDF) [file ppat.1005366.s009.pdf]

Supplemental Table 2: Oligonucleotides used in this study

Primer Name	Sequence (5`-3`)
qRT-PCR	
cFos Promoter E2BS for	AAGCTGGTCTCGAACTCCTG
cFos Promoter E2BS rev	CCATGACTGCCATTCACAAC
cFos Promoter AP1BS -4756 for	GTGGCATGTGTGTGTGTGTG
cFos Promoter AP1BS -4756 rev	TCCACAATTCCTGTGGGACT
cFos Promoter FAP1 -249 for	GAGCAGTTCCCGTCAATCC
cFos Promoter FAP1 -249 rev	AGCATTTCGCAGTTCCTGTC
Alpha-tubulin o.c. for	GCTTCTTGGTTTTCCACAGC
Alpha-tubulin o.c. rev	GGTGGTGAGGATGGAGTTGT
PGK (human) sense	CTGTGGGGGTATTTGAATGG [1]
PGK (human) antisense	CTTCCAGGAGCTCCAAACTG [1]
cFos o.c. for	CTTCCTGTTCCCAGCATCAT
cFos o.c. rev	GGGGTAGGTGAAGACCAAGG
EMSA	
dAP CRPV URR WT sense	GCATGCGATGACACATTACAC
dAP WT CRPV URR comp	CGTACGCTACTGTGTAATGTG
dAP CRPV URR mut sense	GCATGCGACATATGTTACAC
dAP CRPV URR mut comp	CGTACGCTGTATACAATGTG
Cloning	
AP-MMP9 rev	TCGACTGACTCATGACTCATGACTCATGACTCATGACTCATGACTCATGACTCAA
AP-MMP9 for	AGCTTTGAGTCATGAGTCATGAGTCATGAGTCATGAGTCATGAGTCATGAGTCAG
AP-CRE for	AGCTTTGACGTCATGACGTCATGACGTCATGACGTCATGACGTCATGACGTCATGACGTCAG
AP-CRE rev	TCGACTGACGTCATGACGTCATGACGTCATGACGTCATGACGTCATGACGTCATGACGTCAA
dAP-CRPV for	AGCTTTGACACATGACACATGACACATGACACATGACACATGACACATGACACAG
dAP-CRPV rev	TCGACTGTGTCATGTGTCATGTGTCATGTGTCATGTGTCATGTGTCATGTGTCAA
pAP-CRPV for	GCTTTGACTCATGACTCATGACTCATGACTCATGACTCATGACTCATGACTCAG
pAP-CRPV rev	TCGACTGAGTCATGAGTCATGAGTCATGAGTCATGAGTCATGAGTCATGAGTCAA
mdAP1-CRPV for	GCACCCGGTGGCATGCGACATATGTTACACAGTAAGCCTTGAGAG
mdAP1-CRPV rev	CTCTCAAGGCTTACTGTGTAACATATGTCGCATGCCACCGGGTGC
mpAP1-CRPV for	GGTGTGCATGACTGTAAGTATGCGGCCGCCACCGCATGCGGTGTTATG
mpAP1-CRPV rev	CATAACACCGCATGCGGTGGCGGCCGCATACTTACAGTCATGCACACC

Supplemental Table 2 (continued)

Primer Name	Sequence (5`-3`)
shBrd4-1 for	GATCCCCTGAGCACAATCAAGTCTAATTCAAGAGATTAGACTTGATTGTGCTCATTTTTG
shBrd4-2 for	GATCCCCGCGCTTGGAAAACAACTATTTCAAGAGAATAGTTGTTTTCCAAGCGCTTTTG
shMEK1-1 for	GATCCCCGGATCAAGTCCTGAAGAAAttcaagagaTTTCTTCAGGACTTGATCCTTTTTA
shMEK1-2 for	GATCCCCAGCTTATTCACCTGGAGATttcaagagaATCTCCAGGTGAATAAGCTTTTTTA

[1] Reiser, J., Hurst, J., Voges, M., Krauss, P., Munch, P., Iftner, T., and Stubenrauch, F. (2011). High-risk human papillomaviruses repress constitutive kappa interferon transcription via E6 to prevent pathogen recognition receptor and antiviral-gene expression. Journal of virology *85*, 11372-11380.
