# Supplementary material for: Papillomavirus-Associated Tumor Formation Critically Depends on c-Fos Expression Induced by Viral Protein E2 and Bromodomain Protein Brd4
Source: PLoS Pathog. 2016 Jan 4;12(1):e1005366. doi: 10.1371/journal.ppat.1005366 (PMC4699637; doi:10.1371/journal.ppat.1005366)
Supplement: S3 Table — (PDF) [file ppat.1005366.s010.pdf]

Target protein	Company	Order number	Dilution
**Western blot**			
**Gal4-HRP**	Santa Cruz	sc-510	1:1000
**MEK1**	Santa Cruz	sc-6250	1:1500
**GAPDH**	Santa Cruz	sc-20357	1:1000
**HSP90**	Santa Cruz	sc-69703	1:1000
**α-tubulin**	Calbiochem	CP06	1:1500
**HA**	Covance	MMS-101P	1:1000
**c-Fos**	Cell Signaling	#2250	1:1000
**ChIP**			
**Brd4**	[2]		2µl/IP
**HA**	abcam	ab9110	2µl/IP
**c-Fos**	Cell Signaling	#2250	2µl/IP
**FosB**	Santa Cruz	sc48 X	2µl/IP
**cJun**	Santa Cruz	sc1694 X	2µl/IP
**JunB**	Santa Cruz	sc46 X	2µl/IP
**JunD**	Santa Cruz	sc74 X	2µl/IP

[2] Wu, S.Y., Lee, A.Y., Hou, S.Y., Kemper, J.K., Erdjument-Bromage, H., Tempst, P., and Chiang, C.M. (2006). Brd4 links chromatin targeting to HPV transcriptional silencing. Genes & development *20*, 2383-2396.
