# Supplementary material for: Papillomavirus-Associated Tumor Formation Critically Depends on c-Fos Expression Induced by Viral Protein E2 and Bromodomain Protein Brd4
Source: PLoS Pathog. 2016 Jan 4;12(1):e1005366. doi: 10.1371/journal.ppat.1005366 (PMC4699637; doi:10.1371/journal.ppat.1005366)
Supplement: S1 Text — (PDF) [file ppat.1005366.s011.pdf]

Supplemental Experimental Procedures

Plasmids and CRPV constructs

PC18-SP1-Luc reporter plasmid has been previously described [1, 2].
The CRPV-URR reporter plasmid was created by amplifying the region between nt 10805 and 1074 of pLAII-CRPV [3] and insertion into pGL3 basic vector (Promega) via KpnI and NcoI restriction sites. The E7 ATG is in frame with the luciferase ATG. The reporter constructs harboring mutated AP1BS within the CRPV-URR (mdAP, mpAP and mdpAP) were generated via Overlap Extension PCR using CRPV-URR reporter plasmid as template and the primers mdAP1-CRPV for and rev and mpAP1-CRPV for and rev listed in Table S2. The nucleotide sequence of the distal and the proximal AP1BS was replaced with NdeI and NotI restriction sites, respectively.

pSG5-plasmids expressing wt CRPV E2 , CRPV E2 320/321 and E2-I73A have been described [4]. The dominant negative human pcDNA4C-SV40NLS-hBrd4-CTD has been described and was kindly provided by Peter Howley [5]. To construct CRPVE2 320KM/321CR the wildtype nucleic acid sequence AAGTGC encoding K320/C321 was mutated to ATGAGG encoding M320/R321 by overlap extension PCR (forward primer 5` - caaccagcttatgaggttaaggtatc - 3` and reverse primer 5`- gataccttaacctcataagctggttg – 3`).

To generate the artificial AP1-dependent reporters AP-MMP9, AP-CRE, dAP-CRPV and pAP-CRPV, double stranded oligonucleotides (Table S2) were cloned in pMCS-luc vector (Stratagene, La Jolla, USA) using HindIII and SalI restriction sites. The AP1 sites are derived from the human MMP9 promoter, the HPV18 promoter; the CRPV URR or encode a generic cAMP response element (CRE) binding site. Consecutive shortened fragments of the c-fos promoter reporter construct from – 5238 to – 362 relative to the c-fos ATG driving the firefly luciferase gene were constructed with PCR and respective primers.
The pAP-1-Luc reporter, carrying an AP1 sequence identical to the AP1BS located in the HPV18 LCR, and pFCMEKK plasmids are commercially available (Stratagene, La Jolla, USA).

The pLAII-CRPV construct was cloned from a papilloma of a cottontail rabbit and has been described [3]. The pLAII-CRPV-mdAP, pLAII-CRPV-mpAP and pLAII-CRPV-mdpAP constructs were generated via Overlap Extension PCR using pLAII-CRPV as a template and the mdAP1-CRPV for and rev and mpAP1-CRPV for and rev listed in Table S2, digested with BlpI and AvrII restriction enzymes and cloned into BlpI/AvrII digested pLAII-CRPV.

pLAII-CRPVshLuc has previously been described [6]. The pLAII-CRPVshBrd4-1, pLAII-CRPVshBrd4-2, pLAII-CRPVshMEK1-1 and pLAII-CRPVshMEK1-2 genomes were constructed via previous annealing of the several forward and reverse shRNA oligonucleotides (Table S2) and cloning into pSUPER vector [7] through BglII/SalI or BglII/HindIII restriction sites as previously described [6]. To generate pSG-CRPV E2HA, an oligonucleotide was introduced in the SmaI site at nt 3984 in the CRPV E2 coding sequence. pSG CRPV E2HA 320/321 and E2HA I73A were generated by exchanging the C-terminus harbouring the HA tag by digestion with MluI and BglII. For generation of pIRESp CRPV E2HA, CRPV E2HA 320/321 and E2HA I73A the respective pSG-constructs were digested with BamHI and inserted into BamHI digested pIRESpuro3 (Clontech). For generation of

pRTS CRPV E2-HA, CRPV E2-HA was amplified by PCR to add flanking SfiI sites. The amplicon was digested with SfiI and inserted into pRTS1 [8]. All cloned and mutated vectors were confirmed by DNA sequencing.

Cell culture and transient luciferase assay

C33A and 293T cells were cultivated in Dulbecco's modified Eagle Medium (DMEM) (Life Technologies) supplemented with 10% fetal bovine serum and 45µg/ml gentamicin (PAA).
AVS cells [9] were cultured in Keratinocyte serum free medium (KSFM) without antibiotics but supplemented with bovine pituitary extract and human recombinant epidermal growth factor (Life Technologies).

For luciferase assays, approximately $7x10^4$ C33A cells were seeded in 24-well culture plates. After 24 h 50ng of reporter and 5ng of expression plasmids diluted in 25µl OptiMEM (Life Technologies) were cotransfected according to the manufacturer's instructions using a FuGENE®-DNA ratio of 5:1 (Promega) or 2µl of lipofectamine (Life Technologies). Luciferase assays were carried out 48h post-transfection as previously described [1]. Co-transfections were normalized to equal DNA-amounts with the respective empty vector construct.

siRNA transfection

For luciferase assays, C33A cells were transfected 24h after seeding with a pool of three siRNAs against c-Fos (SI02781429, SI02781464, SI00074543, Qiagen) at 60nM final concentration and 10.8µl of HiPerFect reagent (Qiagen) according to the manufacturer's instructions. After 24h the medium was exchanged and DNA was transfected as described above. Luciferase measurements were carried out 48h after DNA transfection. As negative control, 60nM AllStars Negative Control siRNA (No. 1027281 Qiagen) was used. Each measurement was conducted in triplicate and repeated three to five times.

For Western blot analyses, approximately $1.5x10^5$ C33A cells were seeded in 6-well plates and transfected 24h later with either 60nM of the c-Fos siRNA pool, or 60nM Allstar siRNA as negative control using 43.2µl HiPerfect reagent (Qiagen) as described above. The cells were incubated with 10µM MG-132 (Calbiochem) 6h before harvesting in order to prevent protein degradation.
Brd4 siRNA target-sequence was CATGAGCACAATCAAGTCTAA and was custom-made by Qiagen.

Lentiviral infection

$3x10^6$ 293T cells were seeded in 10cm plate and transfected after 24 h with 20µg of pLVTHMshRNA plasmid, 15µg of packaging plasmid (pSPAX2) and 6µg of enveloping plasmid (pMD2.G) (kindly provided by Didier Trono) using the $CaCl_2$-Method. One day after transfection the supernatant containing infectious virus particles was collected and filtered through a 0.45µm PVDF filter (Millex-HV, Millipore). Polybrene was added to a final concentration of 10µg/ml. For transduction of AVS cells, $7x10^4$ cells were seeded out in a 24-well plate 24h before addition of the harvested supernatant.

Quantitative real time PCR

Total RNA was isolated with RNAeasy Kit (Qiagen). For microarray analysis double-stranded cDNA was synthesized from 5 µg of total RNA from selected cell pools using a Superscript choice kit (Invitrogen) with a T7-(dT)24 primer incorporating a T7 RNA polymerase promoter (Metabion). cRNA was prepared and biotin labeled by in vitro transcription (Enzo

Biochemical). Labeled RNA was fragmented by incubation at 94°C for 35 min in the presence of 40 mM Tris–OAc (pH 8.1), 100 mM KOAc and 30 mM MgOAc. Labeled, fragmented cRNA (15 µg) was hybridized for 16 h at 45°C to a GeneChip Human exon 1.0 ST Array (Affymetrix). After hybridization, gene chips were automatically washed and stained. After scanning, the images were subjected to visual inspection and analyzed using the MAS 5 algorithm (Affymetrix). All chips were scaled to an average signal value of 150 to enable comparison between individual arrays. Comparisons were done between cell pools harboring pRTS1-CRPVE2HA or only pRTS1 after induction with doxycycline for 48h. For qPCR cDNA was synthesized from 1µg total RNA using QuantiTect®Reverse Transcription Kit (Qiagen) according to the manufacturers' instructions. The qRT-PCR was conducted with Light®Cycler 480 (Roche) using SYBR Green I Master (Roche), 50ng of cDNA and 3µM forward and reverse primers. PCR was carried out as described [10]. Relative amount of transcripts was calculated using PGK (human) or α-tubulin (rabbit) transcripts as reference [11]. Primer sequences are listed in Table S2.

Microarray Processing and Data Analysis

RNAs were isolated from three independently generated C33A pools harboring pRTS1-CRPVE2-HA in the presence and absence of doxycycline.

For expression profiling using the Affymetrix Human Gene 1.0 Array, 300ng of total RNA was linearly amplified and biotinylated using the WT Labeling Kit (Affymetrix) according to the manufacturer's instructions. Labeled and fragmented cDNA (2.75µg) was hybridized to Human Gene 1.0 Array (Affymetrix). Subsequently arrays were washed and stained in a Fluidics Station 450 (Affymetrix) using the recommended washing procedure. Biotinylated cDNA/cDNA bound to target molecules was detected with streptavidin-coupled phycoerithrine, biotinylated antistreptavidine IgG antibodies and again streptavidine-coupled phycoerithrine according to the protocol. Arrays were scanned using the GCS3000 Gene Chip scanner (Affymetrix) and GCOS 1.4 software. Scanned images were subjected to visual inspection to control for hybridization artifacts and proper grid alignment and analyzed with Expression Console 1.0 (Affymetrix) to generate report files for quality control. For statistical data analysis the CEL-files from the Human Gene 1.0 Array were normalized by RMA algorithms and analysed further by different Bioconductor programs like „affy", „limma", „affyPLM", „GOstats", „genefilter", and „annaffy". Genes that show an at least two-fold increase or decrease in average expression were analyzed in a Welsh's t-test for significant differences and corrected for multiple testing according to Benjamini and Hochberg (BH-FDR).

Immunoblot analyses

Cells were washed and harvested in 1ml cold PBS. After centrifugation, the pellet was resuspended in 4x SDS gel loading buffer (Carl Roth), heated 5 minutes at 95°C and sonificated. The lysate was separated on 10% SDS-PAGE and transferred on a nitrocellulose membrane (Protran; Whatman) in 10 mM N-cyclohexyl-3-amino-propanesulfonic acid (pH 10.3). The blots were blocked 1h with 5% nonfat dry milk in 1x Tris-Buffered Saline (TBS)-0.1% Tween 20 (TBST) at room temperature and then incubated in 5% milk-TBST with the diluted primary antibodies (Table S3) over night at 4°C. Secondary antibodies conjugated to HRP (Dako) were diluted 1:2500 in 5% milk-TBST. Bound antibodies were detected by using SuperSignal West Dura reagent (Thermo Fischer Scientific) and were visualized by use of a Fluor-S MultiImager (Bio-Rad).

Chromatinimmunoprecipitation (ChIP)

Briefly, protein–DNA complexes were crosslinked in vivo by adding formaldehyde to the medium to a final concentration of 1%. The reaction was conducted at room temperature for 10 min and then terminated by the addition of glycine to a final concentration of 0.125 M. Cells were collected by centrifugation at 1000 rpm for 2 min, rinsed twice with ice-cold 1× PBS, resuspended in 1 ml of ice-cold cell lysis buffer (5 mM PIPES, pH 8.0, 85 mM KCl, 0.5% NP-40, 1 mM DTT, 0.25 mM PMSF, plus the protease inhibitors: 1 µg/ml each of pepstatin, leupeptin and aprotinin), and kept on ice for 10 min. Cells were then collected and resuspended in 500 µl of nuclear lysis buffer (50 mM Tris–HCl, pH 8.1, 10 mM EDTA, 1% SDS, 1 mM DTT, 2.5 mM PMSF, and the protease inhibitors mentioned above). Sonication was carried out using a Branson Sonifier 450 (3.2mm tapered Micro Tip, duty cycle 50%, output level 4.5) for six 10s bursts with 1 min on ice between each sonication. The sheared chromatin contains DNA fragments averaging between 200 and 500 bp. For each ChIP, 100 µl of sheared chromatin was diluted to 1 ml with IP dilution buffer (0.01% SDS, 1.1% Triton X-100, 1.2 mM EDTA, 16.7 mM Tris–HCl, pH 8.1 and 167 mM NaCl). After short spinning to remove cellular debris the supernatant was incubated with 1 µl of antibodies (Table S3), or no antibodies (as mock IP) at 4°C overnight. The immune complexes were pulled down by further incubating with 20 µl of magnetic protein A/G–beads (ThermoFisher scientific) at 4°C for 1 h. The beads were then washed sequentially with 0.5 ml of IP dilution buffer, TSE (0.1% SDS, 1% Triton X-100, 2 mM EDTA, 20 mM Tris–HCl, pH 8.1, 500 mM NaCl), LiCl buffer (100 mM Tris–HCl, pH 8.1, 500 mM LiCl, 1% NP-40, 1% deoxycholic acid) and TE (10 mM Tris–HCl, pH 8.1, 1 mM EDTA, pH 8.0), twice in each buffer. Immunocomplexes were eluted twice with 250 µl of 50 mM $NaHCO_3$ with 1% SDS at room temperature for 30 min and pooled together. Cross-links were reversed by heating at 65°C in a water bath overnight with the addition of 5 M NaCl to a final concentration of 200 mM. All the samples were digested with 10 µg each of RNase A at 37°C for 1 h and proteinase K at 55°C for 3 h to remove RNA and protein. The DNA samples were purified by phenol–chloroform extraction, followed by ethanol precipitation, finally dissolved in 200 µl of TE and measured by quantitative real-time-PCR.

Immunohistochemistry

Cryostat skin and papilloma sections were rehydrated in TBS-Tween for a few minutes and incubated overnight at 4°C with c-Fos antibody (#2250; Cell Signaling, Danvers) diluted 1:25 in REAL™ Antibody Diluent (Dako, Hamburg, Germany). The sections were washed with TBS-Tween and incubated with the biotin-SP affinity purified anti-rabbit IgG (711-066-152; Jackson ImmunoResearch Laboratories, Inc., West Grove, PA, USA) at RT for 30 min. The slides were washed with TBS-Tween and incubated with the horseradish peroxidase-conjugated streptavidin at RT for 30 min and washed again. The sections were incubated with 1-2 drops of Liquid DAB Chromogen (BioGenex, San Ramon, CA, USA) for few minutes and rapidly washed with tap water. The sections were counterstained with hematoxylin and mounted with Glycergel (Dako, Hamburg, Germany).

References

1. Behren A, Simon C, Schwab RM, Loetzsch E, Brodbeck S, Huber E, et al. Papillomavirus E2 protein induces expression of the matrix metalloproteinase-9 via the extracellular signal-regulated kinase/activator protein-1 signaling pathway. Cancer Res. 2005;65(24):11613-21. Epub 2005/12/17. doi: 65/24/11613 [pii] 10.1158/0008-5472.CAN-05-2672. PubMed PMID: 16357172.
2. Zobel T, Iftner T, Stubenrauch F. The papillomavirus E8-E2C protein represses DNA replication from extrachromosomal origins. Molecular and cellular biology. 2003;23(22):8352-62. PubMed PMID: 14585992; PubMed Central PMCID: PMC262328.
3. Nasseri M, Meyers C, Wettstein FO. Genetic analysis of CRPV pathogenesis: the L1 open reading frame is dispensable for cellular transformation but is required for papilloma formation. Virology. 1989;170(1):321-5. PubMed PMID: 2541552.
4. Jeckel S, Huber E, Stubenrauch F, Iftner T. A transactivator function of cottontail rabbit papillomavirus e2 is essential for tumor induction in rabbits. J Virol. 2002;76(22):11209-15. Epub 2002/10/22. PubMed PMID: 12388680; PubMed Central PMCID: PMC136747.
5. You J, Croyle JL, Nishimura A, Ozato K, Howley PM. Interaction of the bovine papillomavirus E2 protein with Brd4 tethers the viral DNA to host mitotic chromosomes. Cell. 2004;117(3):349-60. PubMed PMID: 15109495.
6. Leiprecht N, Notz E, Schuetz J, Haedicke J, Stubenrauch F, Iftner T. A novel recombinant papillomavirus genome enabling in vivo RNA interference reveals that YB-1, which interacts with the viral regulatory protein E2, is required for CRPV-induced tumor formation in vivo. American journal of cancer research. 2014;4(3):222-33. PubMed PMID: 24959377; PubMed Central PMCID: PMC4065403.
7. Brummelkamp TR, Bernards R, Agami R. A system for stable expression of short interfering RNAs in mammalian cells. Science. 2002;296(5567):550-3. doi: 10.1126/science.1068999. PubMed PMID: 11910072.
8. Bornkamm GW, Berens C, Kuklik-Roos C, Bechet JM, Laux G, Bachl J, et al. Stringent doxycycline-dependent control of gene activities using an episomal one-vector system. Nucleic Acids Res. 2005;33(16):e137. Epub 2005/09/09. doi: 33/16/e137 [pii] 10.1093/nar/gni137. PubMed PMID: 16147984; PubMed Central PMCID: PMC1201338.
9. Huber E, Vlasny D, Jeckel S, Stubenrauch F, Iftner T. Gene profiling of cottontail rabbit papillomavirus-induced carcinomas identifies upregulated genes directly Involved in stroma invasion as shown by small interfering RNA-mediated gene silencing. Journal of virology. 2004;78(14):7478-89. doi: 10.1128/JVI.78.14.7478-7489.2004. PubMed PMID: 15220421; PubMed Central PMCID: PMC434115.
10. Reiser J, Hurst J, Voges M, Krauss P, Munch P, Iftner T, et al. High-risk human papillomaviruses repress constitutive kappa interferon transcription via E6 to prevent pathogen recognition receptor and antiviral-gene expression. Journal of virology. 2011;85(21):11372-80. doi: 10.1128/JVI.05279-11. PubMed PMID: 21849431; PubMed Central PMCID: PMC3194958.
11. Pfaffl MW. A new mathematical model for relative quantification in real-time RT-PCR. Nucleic acids research. 2001;29(9):e45. PubMed PMID: 11328886; PubMed Central PMCID: PMC55695.
